# Supplementary material for: Effect size and statistical power in the rodent fear conditioning literature – A systematic review
Source: PLoS One. 2018 Apr 26;13(4):e0196258. doi: 10.1371/journal.pone.0196258 (PMC5919667; doi:10.1371/journal.pone.0196258)
Supplement: S1 Data — All extracted and calculated data are present in the .accdb file. Each spreadsheet is connected to the level above it by an automatic field code. The complete description of each variable is presented in the database info PDF file. (ZIP) [file pone.0196258.s001.zip › Database_Info.pdf]

Extraction database information

The database contains 4 levels of information: “Paper” refers to article-level information. “Graph” refers to each figure from which comparisons are obtained. “Comparison” contains the extracted data for each comparison (i.e. experiment-level data). “Protocol” contains information related to the training, testing and animals used in each experiment.

The organization and relationships are applied to the database tables as follows:

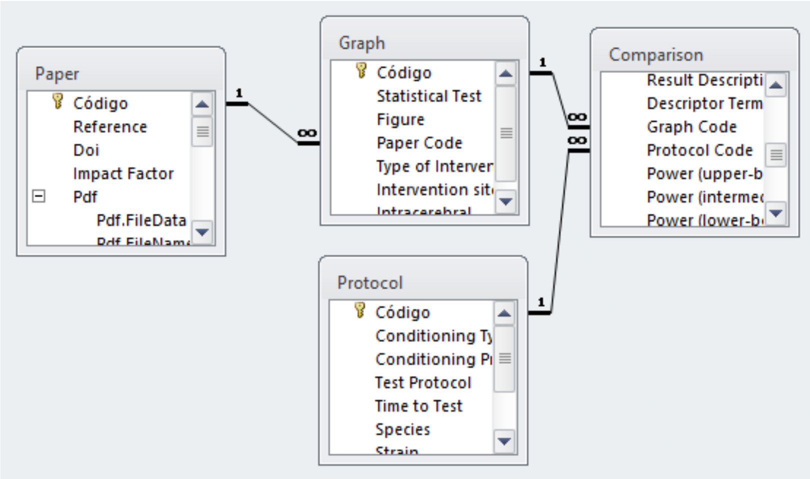

Attributes from the Spreadsheets

<table>
<tr><th colspan="2">Paper Spreadsheet</th></tr>
<tr><th>Attribute</th><th>Description</th></tr>
<tr><td>Reference</td><td>Internal identification for the paper, related to the master search list obtained from PubMed.</td></tr>
<tr><td>DOI</td><td>DOI of the published article.</td></tr>
<tr><td>Journal</td><td>Name of the journal in which the article was published.</td></tr>
<tr><td>Impact factor</td><td>2013 Impact factor for the respective journal.</td></tr>
<tr><td># Citations</td><td>Number of times the article was cited as of August 26th, 2016.</td></tr>
<tr><td>Randomization</td><td rowspan="7">These are the quality assessment items evaluated to assess risk of bias, as described in the Methods session.</td></tr>
<tr><td>Blinding</td></tr>
<tr><td>Sample size calculation</td></tr>
<tr><td>Regulatory requirements</td></tr>
<tr><td>Conflict of Interest</td></tr>
<tr><td>ARRIVE guidelines</td></tr>
<tr><td>Sample size description</td></tr>
</table>

Study quality score	Summarized study quality score (i.e. number of quality assessment items present).
Freezing detection method	Distinguishes whether freezing was scored manually or automatically (including the software used).
Country of origin	Obtained from each paper using the corresponding author's affiliation.
Region of origin	Manually assigned from country of origin information.
% of significant results	Obtained manually from the significance information contained in the comparisons spreadsheet. Note that the percentage refers only to fear conditioning experiments that met our criteria for inclusion, and not for all the experiments present in the paper.

**Graph Spreadsheet**	
**Attribute**	**Description**
Statistical test	Statistical test from which p values were obtained according to the paper.
Figure	The figure of the article that contains the included comparisons.
Paper code	Automatically generated code for each paper included in the database, used as reference for relationships between Graph and Paper.
Type of intervention	States if the intervention used was genetic, pharmacological, surgical or behavioral.
Intervention site	Target region of intervention (i.e. systemic or specific brain region).
Intracerebral	Summarizes if the intervention was applied intracerebrally or not

**Comparison Spreadsheet**	
**Attribute**	**Description**
Control Group	The name each group is given in the figure.
Treated Group	
Intervention target	Molecular or behavioral target of the given intervention.
Control Freezing	Contain each group's mean freezing value, extracted from figures or text.
Treated Freezing	
Control SEM	Contain the respective standard error of the mean, usually extracted from figures or text.
Treated SEM	
Control Sample Size	Number of animals in each group (if not clearly stated in the article, this field was left blank).
Treated Sample Size	
Control SD	Standard deviation, calculated from the SEM and sample size information obtained for each group, or directly from the data itself when available.
Treated SD	
Significant	Refers to whether the comparison was found to be significant at $p<0.05$ according to the statistical test used in the paper.

p value	p value of the comparison as available in the text or figures (i.e. either exact or as greater/smaller than a threshold value).
Pooled SD	Calculated using both groups' sample size and SD values as $\frac{\sqrt{((\mathrm{nControl}-1)\times(\sigma\mathrm{Control}^2))+((\mathrm{nTreated}-1)\times(\sigma\mathrm{Treated}^2))}}{\sqrt{\mathrm{nControl}+\mathrm{nTreated}-2}}$
Effect Size (in Cohen's D)	Effect size expressed as the number of pooled standard deviations between groups.
Effect Size (in %)	Effect size expressed as percentage of the control group's freezing levels.
Result description	"Result description" contains the whole sentence in which the result of each comparison is described in the text or legend. The actual descriptor term used in the survey is included in "Descriptor Term", while "Description Score" was calculated on the basis of obtained responses (see Methods and Supplementary Tables S1 and S2).
Descriptor Term	
Description Score	
Graph Code	Are used to include the automatically generated code in each of the Graph and Protocol tables and define their relationship with the Comparison table.
Protocol Code	
Power (upper-bound ES)	Contain the results from the power calculations for each comparison for three different effect sizes, as described in the Methods section, calculated as follows (in which φ is the standard normal distribution function, α is the significance level set at 0.05, σ is the pooled SD) $$\mathrm{Power} = \varphi\left(z - z_{1-\alpha/2}\right) + \varphi\left(-z - z_{1-\alpha/2}\right)$$ $$z = \frac{\mu\mathrm{Control} - \mu\mathrm{Treated}}{\sigma\sqrt{\frac{1}{\mathrm{nControl}} + \frac{1}{\mathrm{nTreated}}}}$$
Power (intermediate)	
Power (lower-bound ES)	
Normalized Effect Size	Effect size expressed as percentage of the higher freezing value between both groups.
Weighted Mean (Control+Treated)	Mean of both groups weighted by sample size, calculated as follows: $$\frac{(\mathrm{nControl}\times\mu\mathrm{Control}) + (\mathrm{nTreated}\times\mu\mathrm{Treated})}{\mathrm{nControl} + \mathrm{nTreated}}$$
Pooled Coefficient of Variation	Calculated as pooled SD / pooled mean.
Contextual Baseline Freezing (Control)	Mean freezing levels for pre-conditioning freezing values in training sessions without tone for the control and treated groups and their respective SEM.
Contextual Baseline Freezing (Treated)	
Contextual Baseline SEM (Control)	
Contextual Baseline SEM (Treated)	
Tone Baseline (Control)	Mean freezing levels for pre-conditioning freezing values in training sessions with tone for the control and treated groups and their respective SEM.
Tone Baseline (Treated)	
Tone Baseline SEM (Control)	
Tone Baseline SEM	

(Treated)	
Effect Sizes (absolute differences)	Effect size expressed as the absolute difference between treated and control groups.
Power (upper-bound, absolute)	Contains the results of statistical power calculations, as described above for three different effect sizes expressed as absolute differences.
Power (intermediate, absolute)	
Power (lower-bound, absolute)	
Power (d = 0.2)	Contains the results of statistical power calculations performed for three different Cohen's d values.
Power (d = 0.5)	
Power (d = 0.8)	

**Protocol Spreadsheet**	
**Attribute**	**Description**
Conditioning Type	Refers to whether the memory was tested for context or cue conditioning.
Conditioning Protocol	Contains the summarized training procedures.
Test Protocol	Contains the summarized testing procedures.
Time to test	Refers to the interval between training and testing.
Species	Description of the animals used in the experiment.
Strain	
Sex	
Intervention time	Time of the intervention, expressed using the training session as reference.
Post-training	Summarizes whether the intervention was post- or pre-training
