# Supplementary figures and images for: Effect size and statistical power in the rodent fear conditioning literature – A systematic review
Source: PLoS One. 2018 Apr 26;13(4):e0196258. doi: 10.1371/journal.pone.0196258 (PMC5919667; doi:10.1371/journal.pone.0196258)

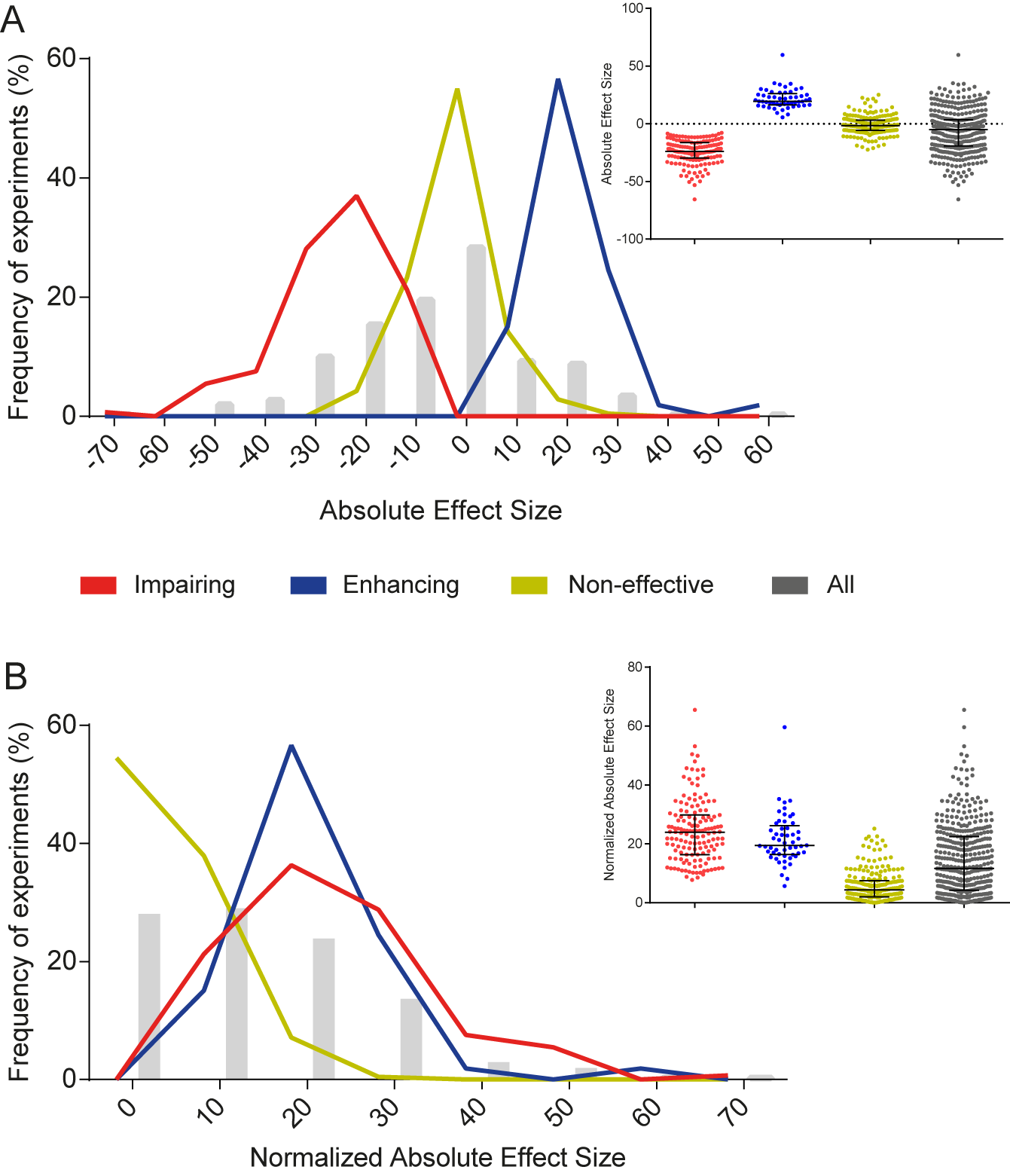

Supplement: S1 Fig — (A) Distribution of effect sizes for experiments, expressed as the absolute difference in freezing between groups. Interventions were divided into memory-impairing (-24.4 ± 10.6, n = 146), memory-enhancing (21.6 ± 8.6, n = 53) or non-effective (-1.09 ± 7.7, n = 211) for graphical purposes, according to the statistical significance of the comparison as informed by authors. Additionally, the whole sample is shown in grey (-6.5 ± 17.7 [-8.2 to -4.7], n = 410). Values are expressed as mean ± SD [95% confidence interval]. Line and whiskers in the inset express median and interquartile interval. (B) Distributions of normalized effect sizes for articles, calculated as the absolute differences between groups. Interventions were divided into memory-impairing (24.4 ± 10.6, n = 146), memory-enhancing (21.6 ± 8.6, n = 53) or non-effective (5.8 ± 5.2, n = 211). Additionally, the whole sample of experiments is shown in grey (14.5 ± 12.0 [13.3 to 15.6], n = 410). (TIF) [file pone.0196258.s002.tif]

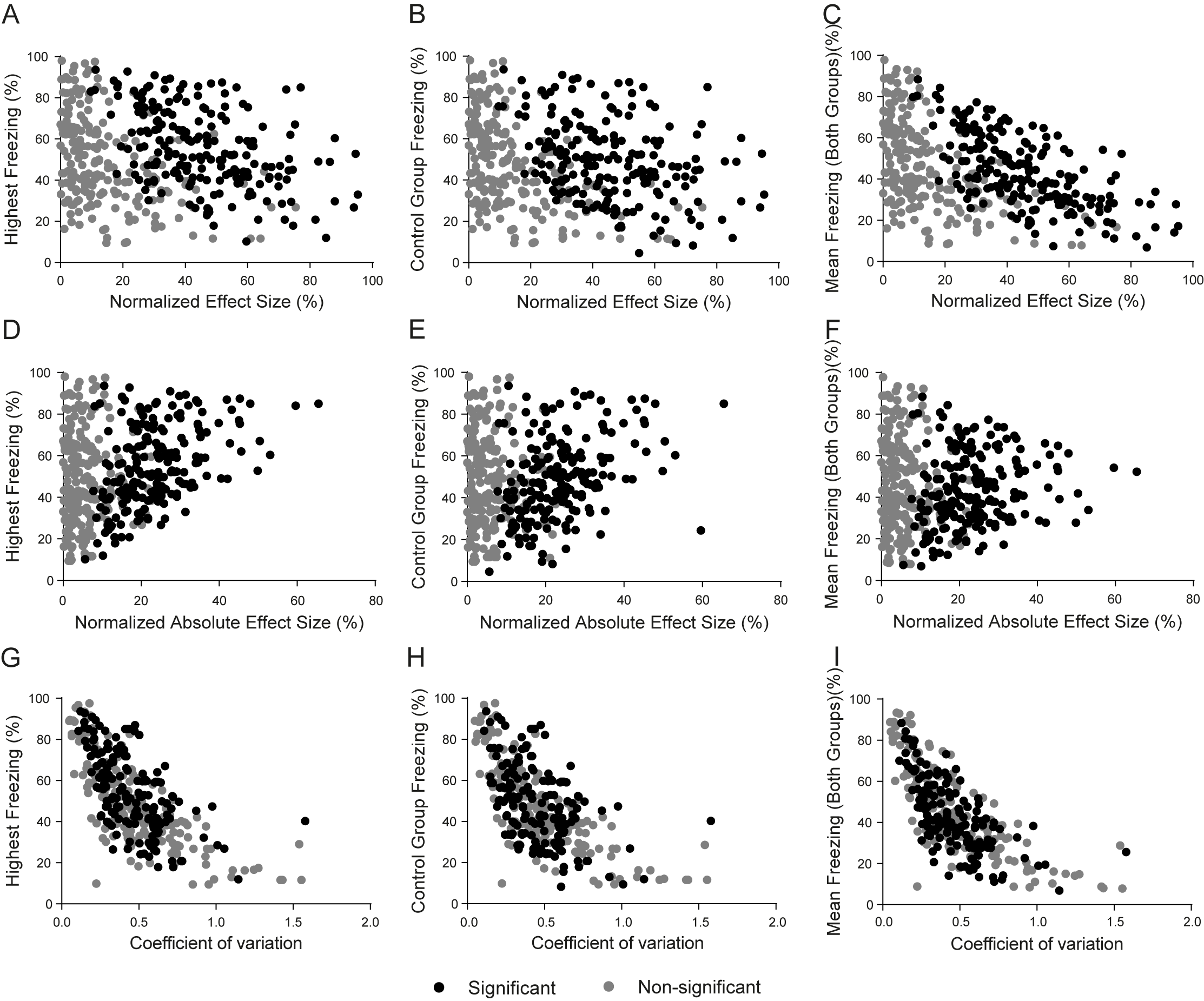

Supplement: S2 Fig — (A) Correlation between the highest mean freezing between both groups and relative effect size, normalized as the % of freezing levels in the highest group. r = -0.20, p<0.0001* (n = 410). (B) Correlation between mean freezing in the control group and normalized effect size. r = -0.25, p<0.0001*. (C) Correlation between mean freezing between groups and normalized effect size. r = -0.45, p<0.0001*. (D) Correlation between the highest mean freezing between both groups and effect size expressed as absolute difference in freezing between groups. r = 0.28, p<0.0001*. (E) Correlation between mean freezing in the control group and absolute effect size. r = 0.18, p = 0.0003*. (F) Correlation between mean freezing between groups and absolute effect size. r = -0.01, p = 0.81. (G) Correlation between highest mean freezing and coefficient of variation (sample size-weighted pooled standard deviation/pooled mean) of both groups. r = -0.69, p<0.0001* (n = 336, as only experiments with exact sample sizes were used to calculate coefficients of variation). (H) Correlation between mean freezing in the control group and coefficient of variation. r = -0.65, p<0.0001*. (I) Correlation between mean freezing between groups and coefficient of variation. r = -0.72, p<0.0001*. Asterisks indicate significant results according to Holm-Sidak correction for 28 experiment-level correlations. (TIF) [file pone.0196258.s003.tif]

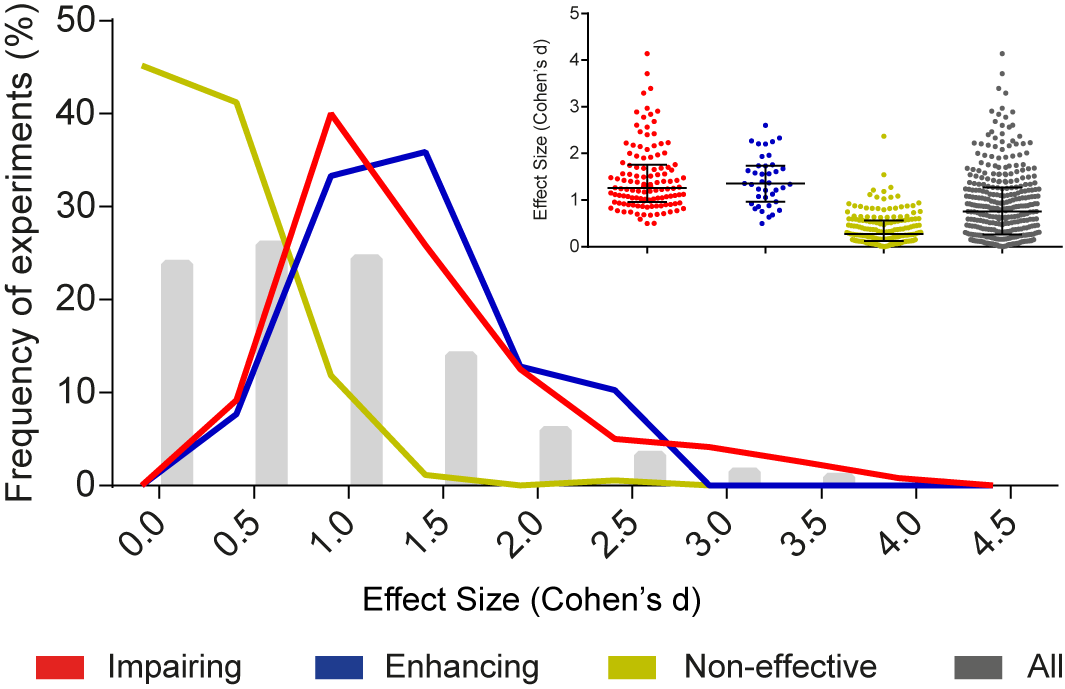

Supplement: S3 Fig — Distribution of effect sizes, expressed as standardized mean differences calculated on the basis of pooled standard deviations (i.e. Cohen’s d). Interventions were divided into memory-impairing (1.5 ± 0.7, n = 120), memory-enhancing (1.4 ± 0.5, n = 39) or non-effective (0.4 ± 0.3, n = 177) for graphical purposes, according to the statistical significance of the comparison as informed by authors. Additionally, the whole sample of experiments is shown in grey (0.9 ± 0.7 [0.8 to 1.0], n = 336). Values are expressed as mean ± SD [95% confidence interval]. Line and whiskers in the inset express median and interquartile interval. (TIF) [file pone.0196258.s004.tif]

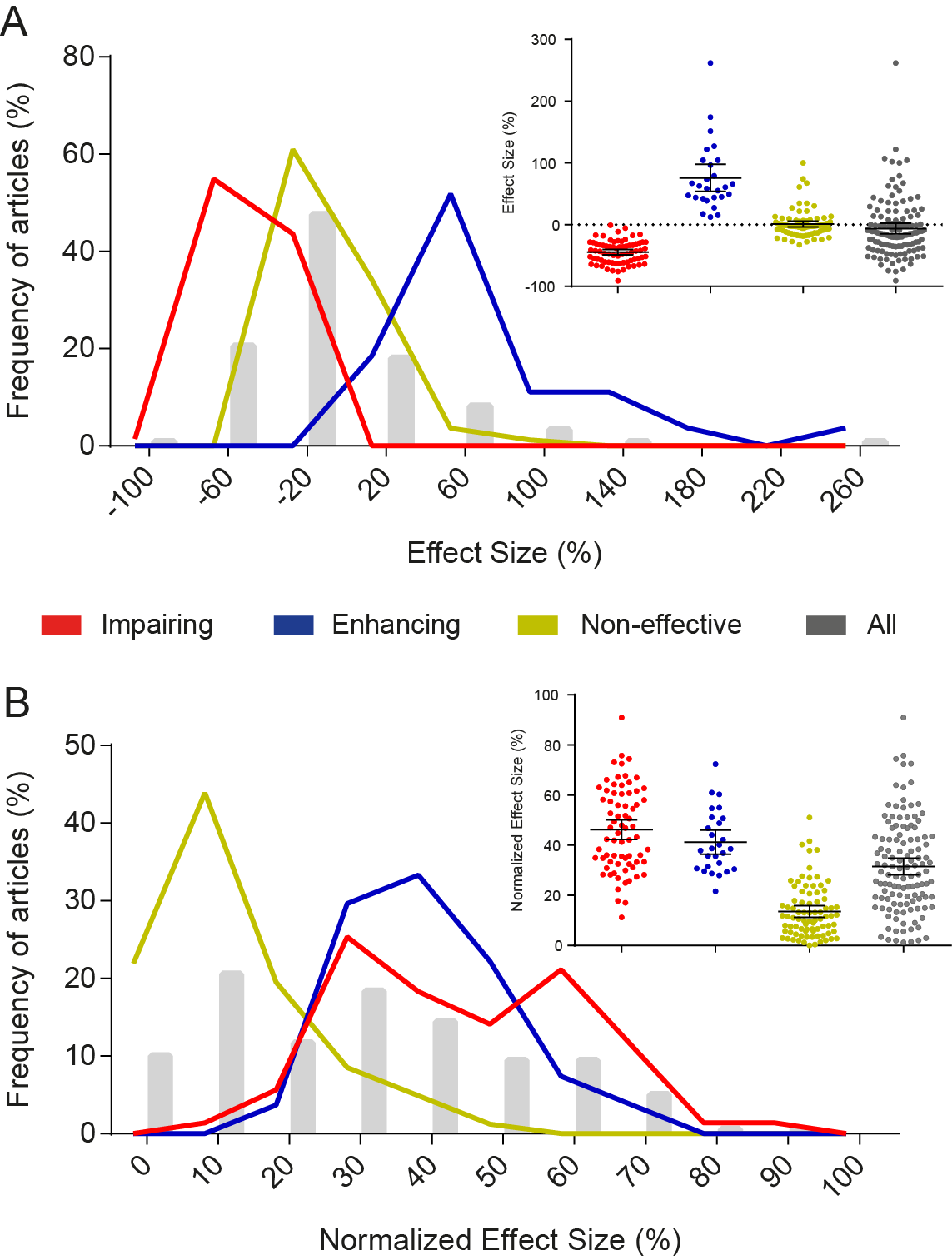

Supplement: S4 Fig — (A) Distribution of mean effect sizes for articles, calculated as percentage of control group freezing. The mean effect size for each type of experiment for each article is shown in the figure–thus, each article can contribute to more than one category if it contains more than one type of experiment (although they are only counted once in the “all” columns). Interventions were divided into memory-impairing (-44.5 ± 18.6%, n = 71), memory-enhancing (75.9 ± 55%, n = 27) or non-effective (1.2 ± 21.8, n = 82). Additionally, the whole sample of articles is shown in grey (-6.1 ± 48% [-14.8 to 2.5], n = 122). Line and whiskers in the inset express median and interquartile interval. (B) Distributions of mean normalized effect sizes for articles, calculated as the percentage of the group with the highest mean. Interventions were divided into memory-impairing (46.2 ± 16.7%, n = 71), memory-enhancing (41.2 ± 12.2%, n = 27) or non-effective (13.6 ± 10.6, n = 82). Additionally, the whole sample of articles is shown in grey (31.5 ± 18.4% [28.2 to 34.8], n = 122). Values are expressed as mean ± SD [95% confidence interval]. (TIF) [file pone.0196258.s005.tif]

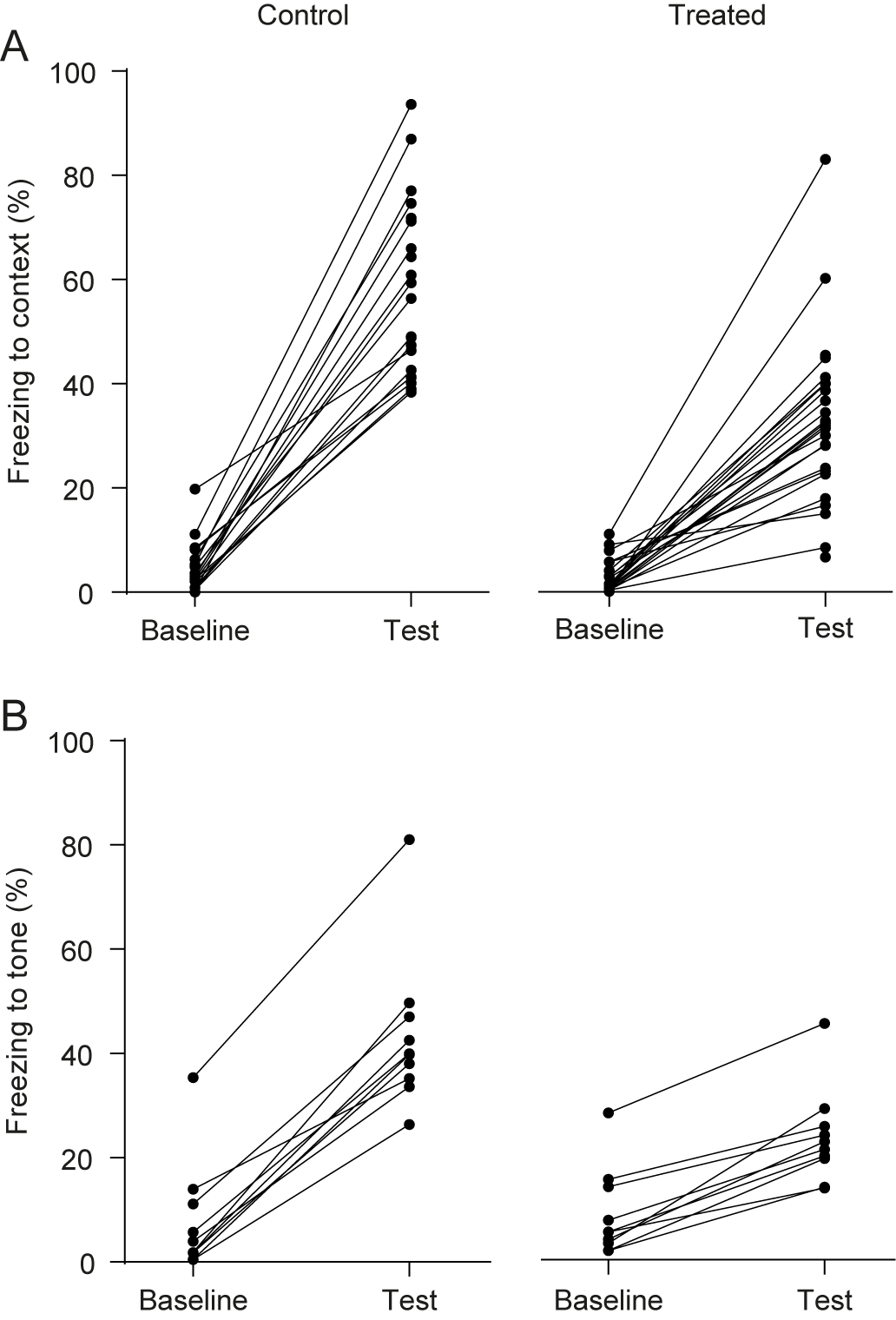

Supplement: S5 Fig — (A) Mean freezing (%) in response to context during training and testing for control (left) and treated (right) groups (n = 25 experiments for which baseline freezing to context–i.e. freezing percentage before any footshocks were applied–was reported for the training session). (B) Mean freezing (%) in response to the first tone during training and in response to the average of all tones during testing, for control (left) and treated (right) animals (n = 10 experiments for which baseline freezing to tone–i.e. freezing percentage to the first tone before pairing with footshock–was reported for the training session). Mean freezing is higher in the test than in the training session in all 35 experiments, with p < 0.05 in 25 (78%) out of the 32 cases in which there was enough information (e.g. exact sample size) to perform an unpaired t test between sessions. (TIF) [file pone.0196258.s006.tif]

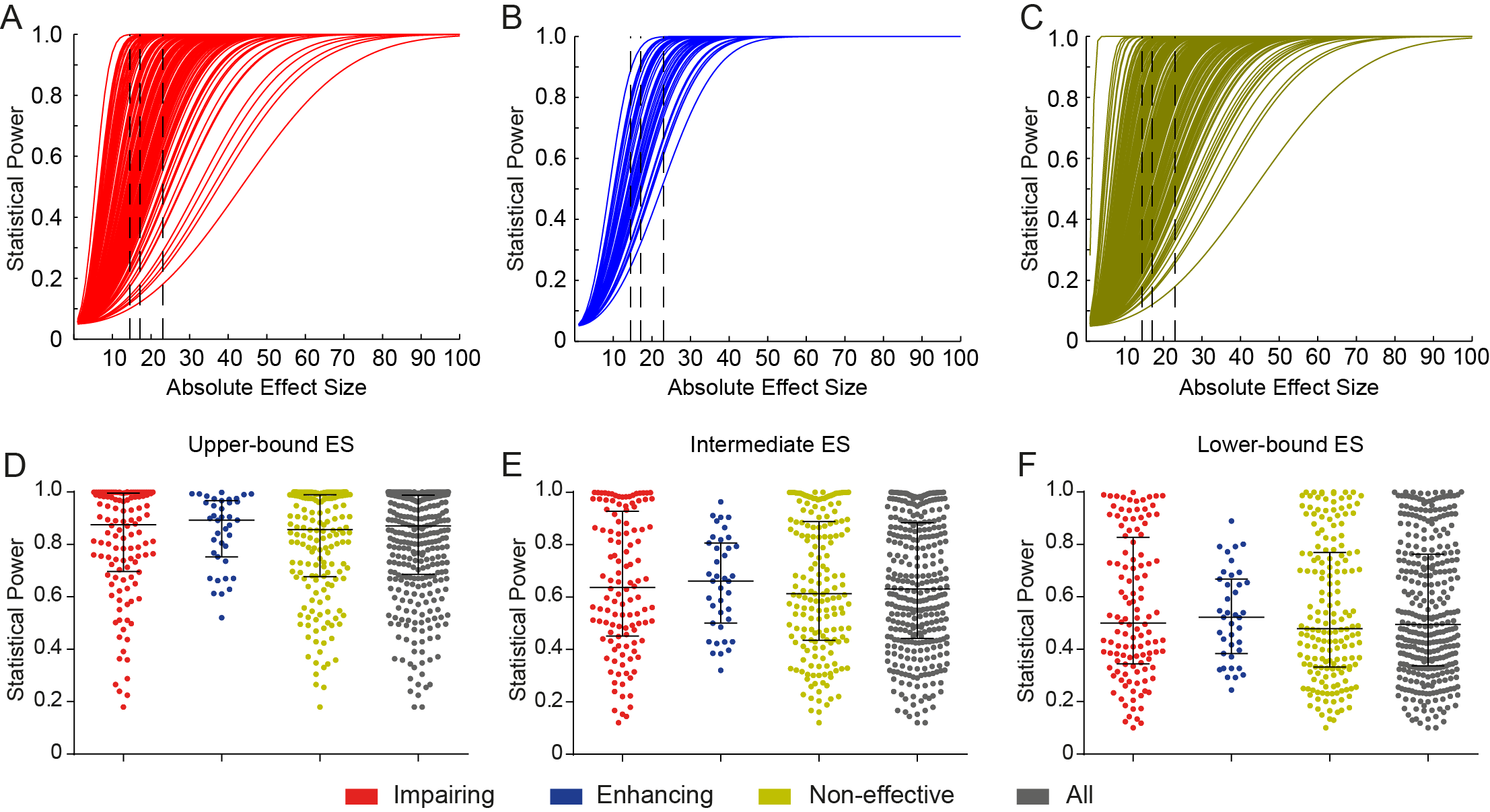

Supplement: S6 Fig — (A) Distribution of statistical power for memory-impairing interventions: based on each experiment’s variance and sample size, power varies according to the absolute difference to be detected for α = 0.05. Dashed lines show the three effect sizes used for point estimates of power in D, E and F. (B) Distribution of statistical power for memory-enhancing interventions. (C) Distribution of statistical power for non-effective interventions. (D) Distribution of statistical power to detect the upper-bound absolute effect size of 23.0% (i.e. mean of statistically significant experiments; right dashed line on A, B and C) for impairing (red), enhancing (blue), non-significant (yellow) and all (grey) experiments. Lines and whiskers express median and interquartile interval. (E) Distribution of statistical power to detect the intermediate absolute effect size of 17.1% (i.e. mean of significant experiments powered at 95% in the analysis described in D; middle dashed line on A, B and C). (F) Distribution of statistical power to detect the lower-bound absolute effect size of 14.5% (i.e. mean of all experiments; left dashed line on A, B and C). (TIF) [file pone.0196258.s007.tif]

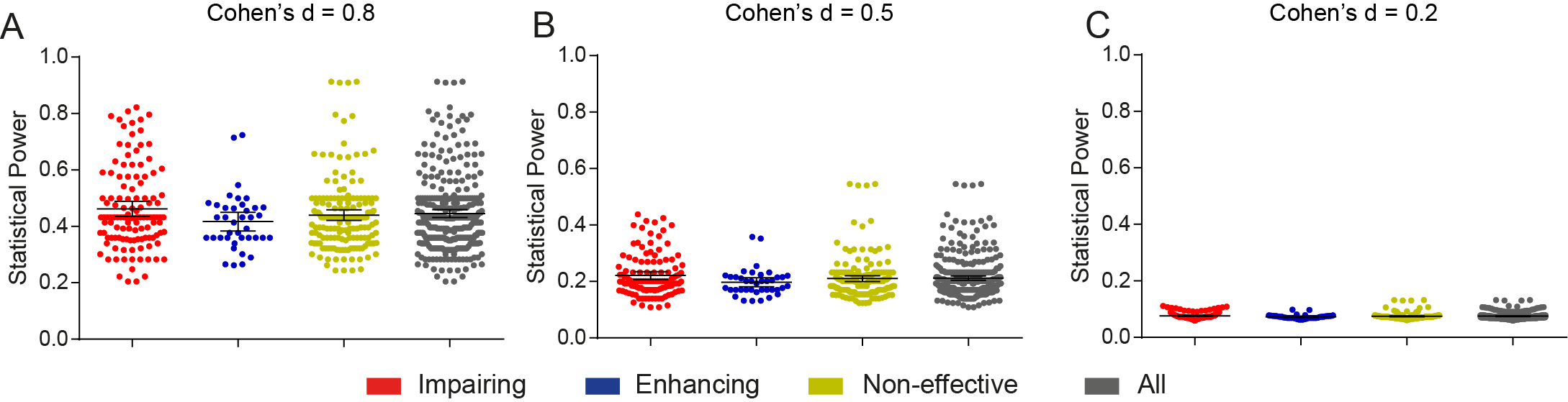

Supplement: S7 Fig — (A) Distribution of statistical power calculated for Cohen’s d = 0.8 (i.e. large effects). for memory-impairing (red, 0.46 ± 0.15, n = 120), memory-enhancing (blue, 0.42 ± 0.10, n = 39), non-effective (yellow, 0.44 ± 0.12, n = 177) and all (grey, 0.44 ± 0.13, n = 336) experiments. (B) Distribution of statistical power calculated for Cohen’s d = 0.5. for memory-impairing (0.22 ± 0.07), memory-enhancing, (0.20 ± 0.05) non-effective (0.21 ± 0.07) and all experiments, (0.21 ± 0.07). (C) Distribution of statistical power calculated for Cohen’s d = 0.2 for memory-impairing (0.08 ± 0.01), memory-enhancing (0.07 ± 0.01), non-effective (0.07 ± 0.01) and all experiments, (0.08 ± 0.01). Lines and whiskers represent median and interquartile interval. (TIF) [file pone.0196258.s008.tif]

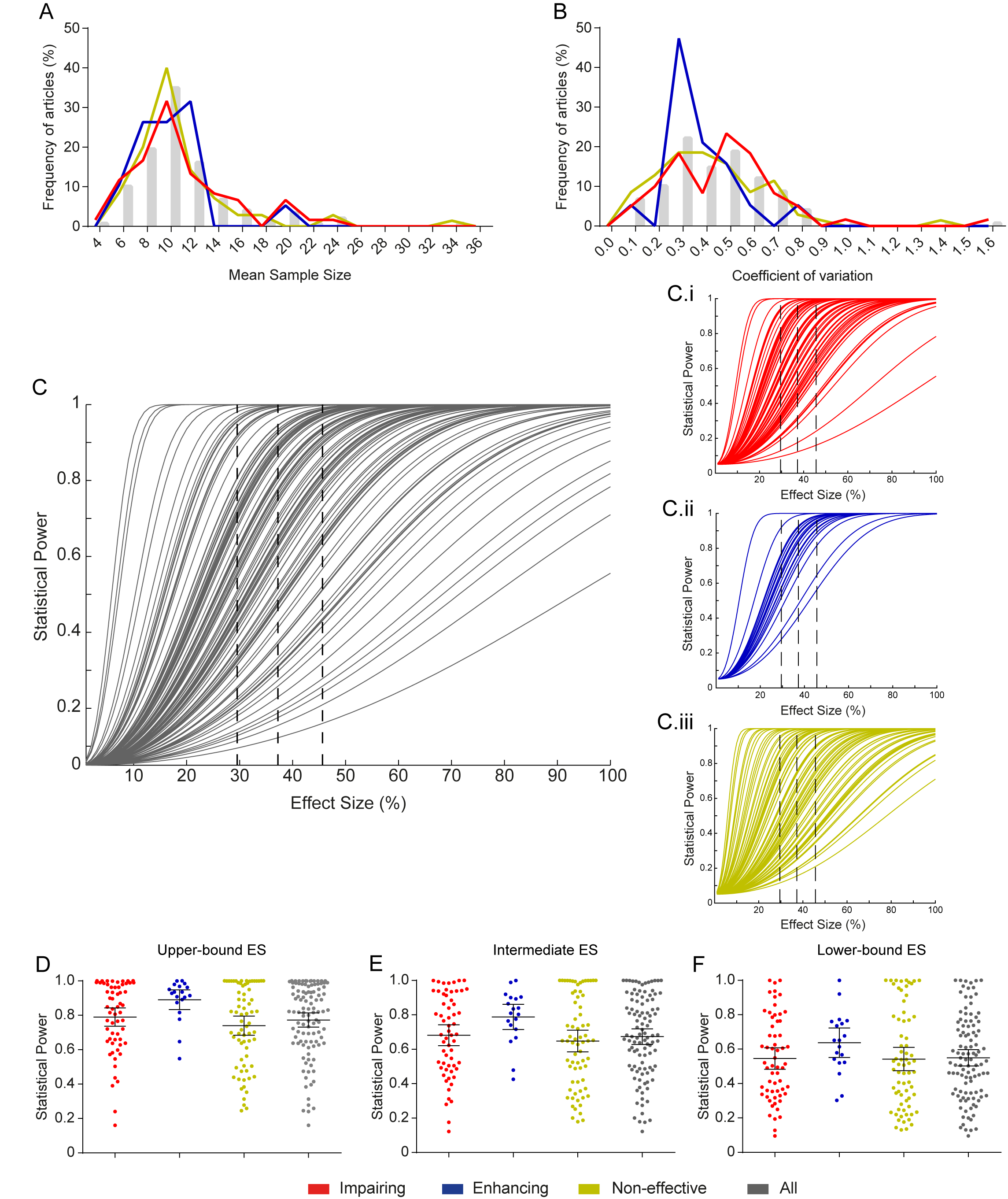

Supplement: S8 Fig — (A) Distribution of mean sample size within articles. Gray bars show the distribution of mean sample size for the whole sample of articles (10.8 ± 4.2 [10.1–11.4], n = 149), while lines show the distribution of the mean sample size of memory-impairing (red, 11 ± 4.2, n = 60), memory-enhancing (blue, 10.1 ± 3.1, n = 19) and non-effective (yellow, 10.7 ± 4.4, n = 70) interventions within each article. Each article can contribute to more than one category if it contains more than one type of experiment, although they are counted only once in the full distribution. Values are expressed as mean ± SD [95% confidence interval]. (B) Frequency distribution of mean coefficients of variation for the whole sample of articles (0.44 ± 0.23 [0.41–0.48], n = 149) and for memory-impairing (0.49 ± 0.23, n = 60), memory-enhancing (0.38 ± 0.14, n = 19) and non-effective interventions (0.42 ± 0.23, n = 70) within each article. (C) Statistical power distribution across articles. Based on each experiment’s variance and sample size, mean power varies according to the difference to be detected for α = 0.05. C.i, C.ii and C.iii show the distribution of statistical power curves for memory-impairing, memory enhancing and non-effective interventions within articles, respectively. Vertical dotted lines mark the effect sizes estimates used for the power calculations in D, E and F. (D) Statistical power calculated based on the upper-bound effect size of 45.6% for the mean of memory-impairing, memory-enhancing and non-effective interventions (using the mean power for each type of experiment in each article) or for all experiments in each article. Line and whiskers express median and interquartile interval. Mean statistical power is 0.77 ± 0.21 [0.73–0.81] (n = 105) for the whole sample, 0.79 ± 0.21 (n = 60) for memory-impairing, 0.89 ± 0.12 (n = 19) for memory-enhancing and 0.74 ± 0.23 (n = 70) for non-effective interventions. (E) Same as D, but using the intermediate effect size of 37.2% for calcu [file pone.0196258.s009.tif]

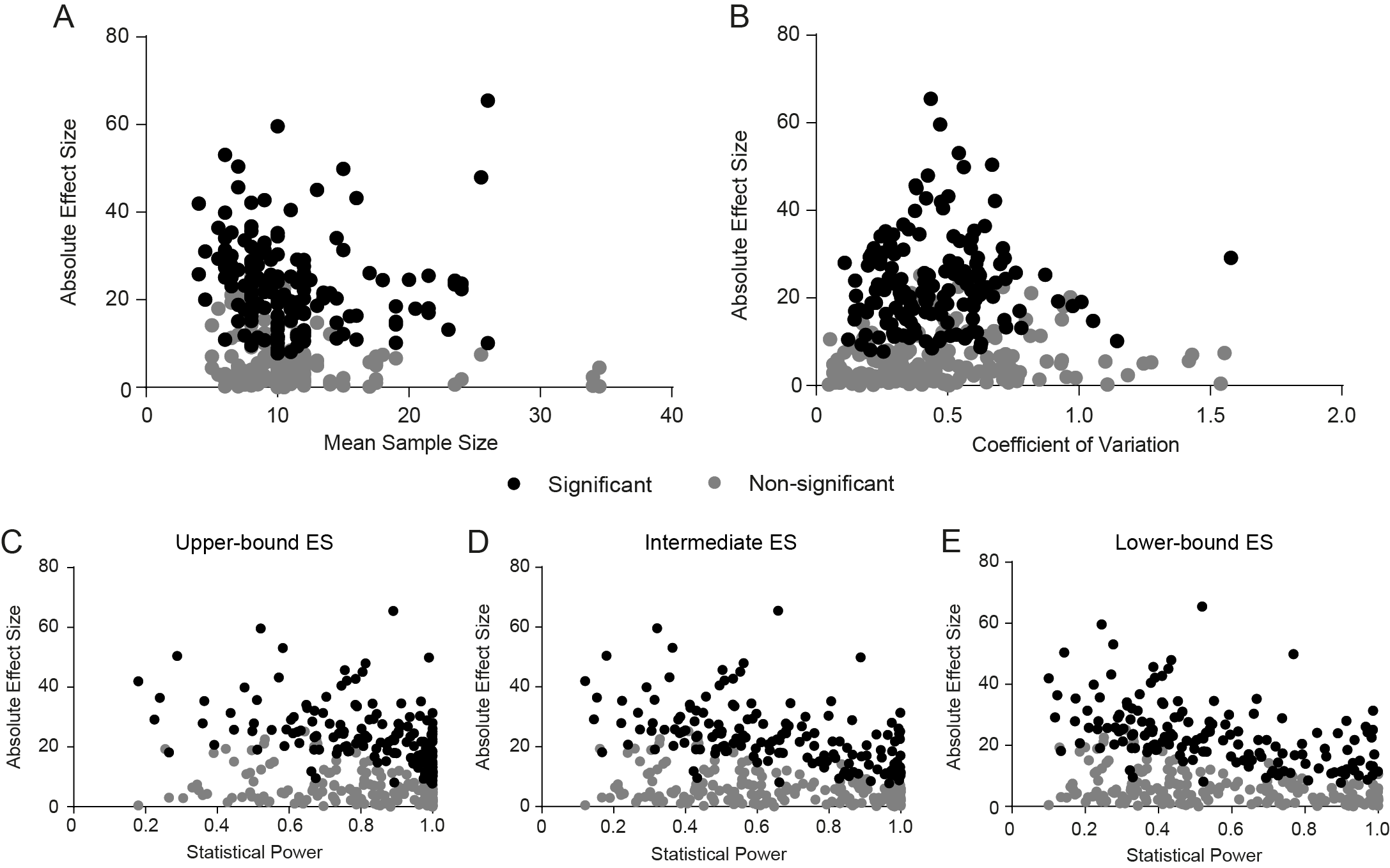

Supplement: S9 Fig — (A) Correlation between absolute effect size and mean sample size. No significant correlation is found (r = -0.05, p = 0.34), although this is largely due to the presence of two outliers. (B) Correlation between absolute effect size and coefficient of variation. Correlation of the whole sample of experiments yields r = 0.02, p = 0.70 (n = 336). (C) Correlation between absolute effect size and statistical power based on upper-bound effect size of 23.0%. Correlation of the whole sample of experiments yields r = -0.24, p<0.0001*. (D) Correlation between absolute effect size and statistical power based on intermediate effect size of 17.1%; r = -0.27, p<0.0001*. (E) Correlation between absolute effect size and statistical power based on lower-bound effect size of 14.5%; r = -0.28, p<0.0001*. Asterisks indicate significant results according to Holm-Sidak correction for 28 experiment-level correlations. (TIF) [file pone.0196258.s010.tif]

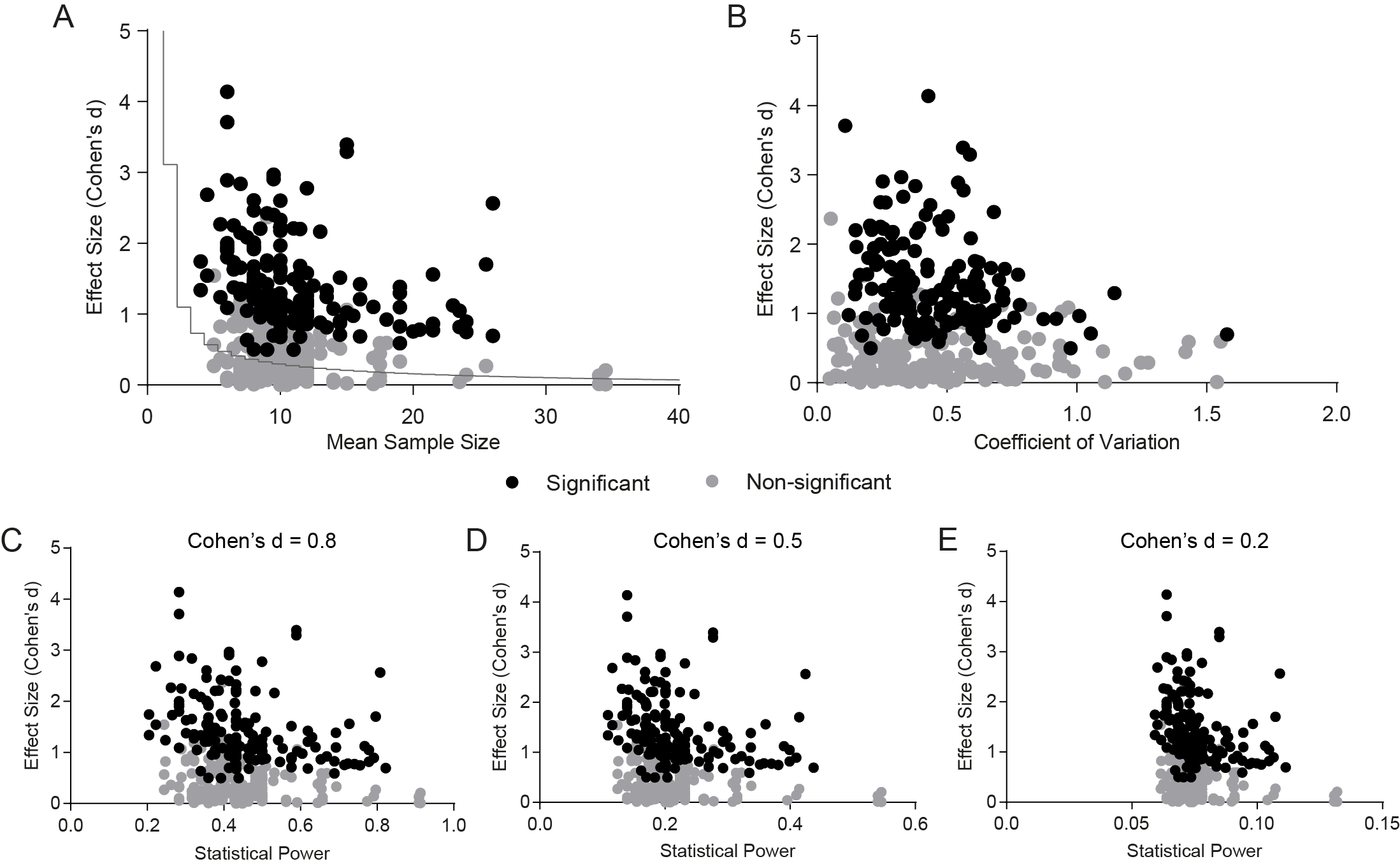

Supplement: S10 Fig — (A) Correlation between effect size expressed in Cohen’s d and mean sample size. Correlation of the whole sample of experiments yields r = -0.15, p = 0.007, suggesting the presence of effect size inflation and/or publication bias. Grey line indicates the effect size needed to achieve p < 0.05 in a two-sample t test with equal numbers of animals in both groups for each sample size. Although significant results are invariably above the line, non-significant results can be observed on both sides due to the use of different statistical tests (e.g. post-hoc tests corrected for multiple comparisons) or different sample sizes between groups (B) Correlation between Cohen’s d and coefficient of variation. The negative correlation (r = -0.14, p = 0.01) is mathematically expected, as higher coefficients of variation will lead effect sizes to be smaller when expressed as Cohen’s d. (C) Correlation between Cohen’s d and statistical power to detect an effect size of d = 0.8. Correlation of the whole sample of experiments yields r = -0.16, p = 0.0025*. (D) Correlation between Cohen’s d and statistical power based on effect size of d = 0.5; r = -0.15, p = 0.0045. (E) Correlation between Cohen’s d and statistical power based on effect size of d = 0.2; r = -0.15, p = 0.0047. Asterisks indicate significant results according to Holm-Sidak correction for 28 experiment-level correlations. (TIF) [file pone.0196258.s011.tif]

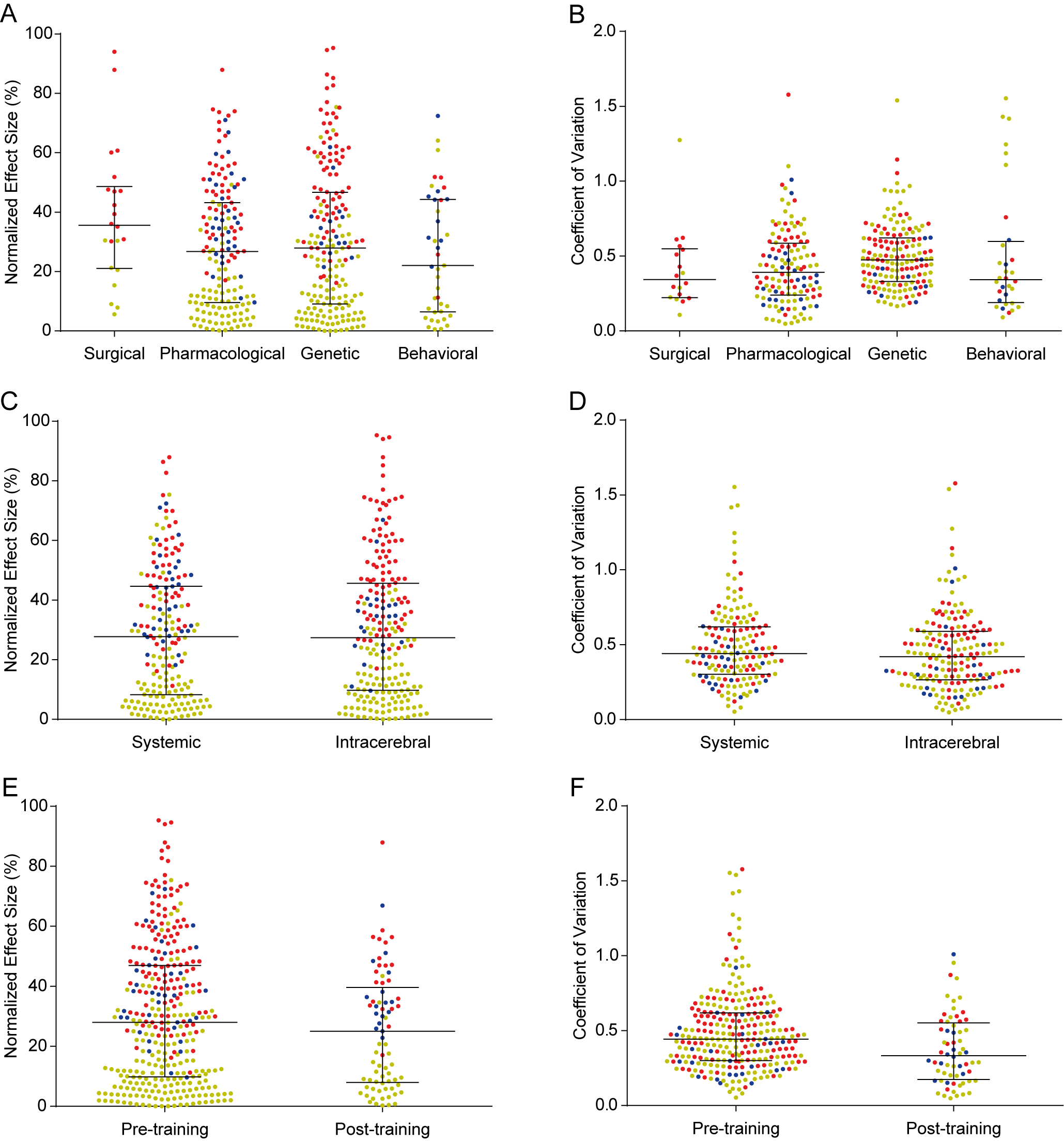

Supplement: S11 Fig — Colors indicate memory-enhancing (red), memory-impairing (blue) or non-effective (yellow) experiments, all of which are pooled together in the analysis. Line and whiskers express median and interquartile interval. (A) Distribution of effect sizes across surgical (n = 22), pharmacological (n = 159), genetic (n = 188) and behavioral (n = 41) interventions. One-way ANOVA, p = 0.12. (B) Coefficients of variation across surgical (n = 18), pharmacological (n = 128), genetic (n = 158) and behavioral (n = 32) interventions. One-way ANOVA, p = 0.08. (C) Distribution of effect sizes across systemic (n = 194) and intracerebral (n = 216) interventions. Student’s t test, p = 0.45. (D) Coefficients of variation across systemic (n = 157) and intracerebral (n = 179) interventions. Student’s t test, p = 0.15. (E) Distribution of effect sizes across interventions applied pre- (n = 333) or post-training (n = 77). Student’s t test, p = 0.07. (F) Coefficients of variation across interventions applied pre- (n = 272) or post-training (n = 64). Student’s t test, p = 0.0015*. For all coefficient of variation analyses, 74 experiments were excluded due to lack of information on sample size for individual groups. Asterisks indicate significant results according to Holm-Sidak correction for 14 experiment-level comparisons. (TIF) [file pone.0196258.s012.tif]

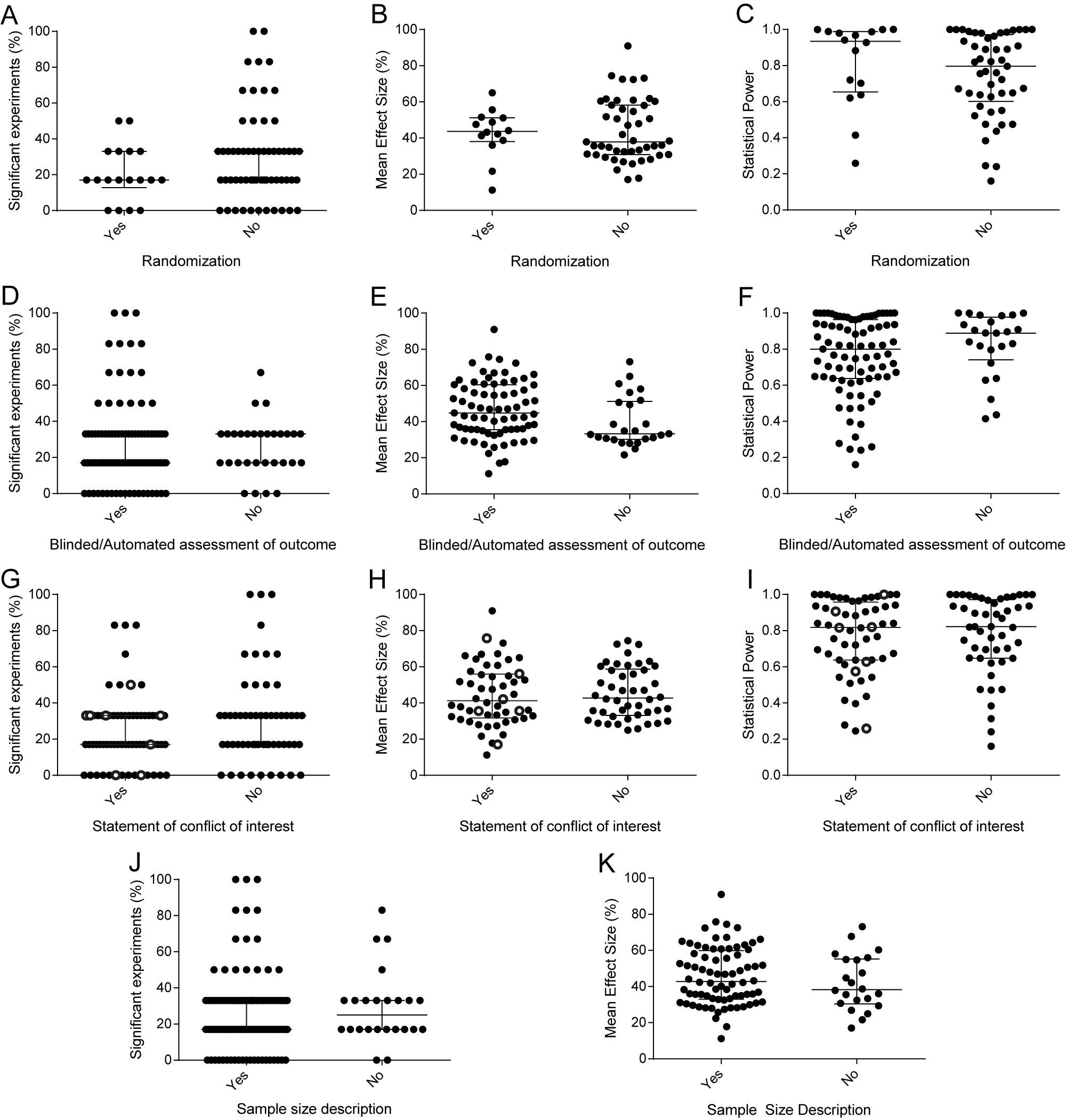

Supplement: S12 Fig — (A) Percentage of significant results in articles with (n = 18) and without (n = 59) randomization. Line and whiskers express median and interquartile interval. Student’s t test, p = 0.13. (B) Mean normalized effect size of significant results in articles with (n = 14) and without (n = 47) randomization. Student’s t test, p = 0.79. (C) Mean statistical power (upper-bound estimate) in articles with (n = 16) and without (n = 49) randomization. Student’s t test, p = 0.33. (D) Percentage of significant results in articles with (n = 92) and without (n = 30) blinded/automated assessment of freezing. Student’s t test, p = 0.65. (E) Mean normalized effect size of significant results in articles with (n = 73) and without (n = 25) blinded/automated assessment. Student’s t test, p = 0.06. (F) Mean statistical power in articles with (n = 81) and without (n = 24) blind/automated assessment. Student’s t test, p = 0.17. (G) Percentage of significant results in articles with (n = 66) and without (n = 56) statement of conflict of interest. Student’s t test, p = 0.12. (H) Mean normalized effect size of significant results in articles with (n = 52) and without (n = 46) statement of conflict of interest. Student’s t test, p = 0.72. (I) Mean statistical power in articles with (n = 56) and without (n = 49) statement of conflict of interest. Student’s t test, p = 0.78. (J) Percentage of significant results per article with (n = 98) and without (n = 24) exact sample size description for fear conditioning experiments. Student’s t test, p = 0.63. (K) Mean normalized effect size of significant results for articles with (n = 76) and without (n = 22) sample size description. Student’s t test, p = 0.33. Sample size varies for each of the three variables, as not all papers have significant results or exact sample sizes allowing power calculations. On panels G-I, white circles indicate papers with a conflict of interest stated, while black circles indicate papers that stated no conflict of interes [file pone.0196258.s013.tif]

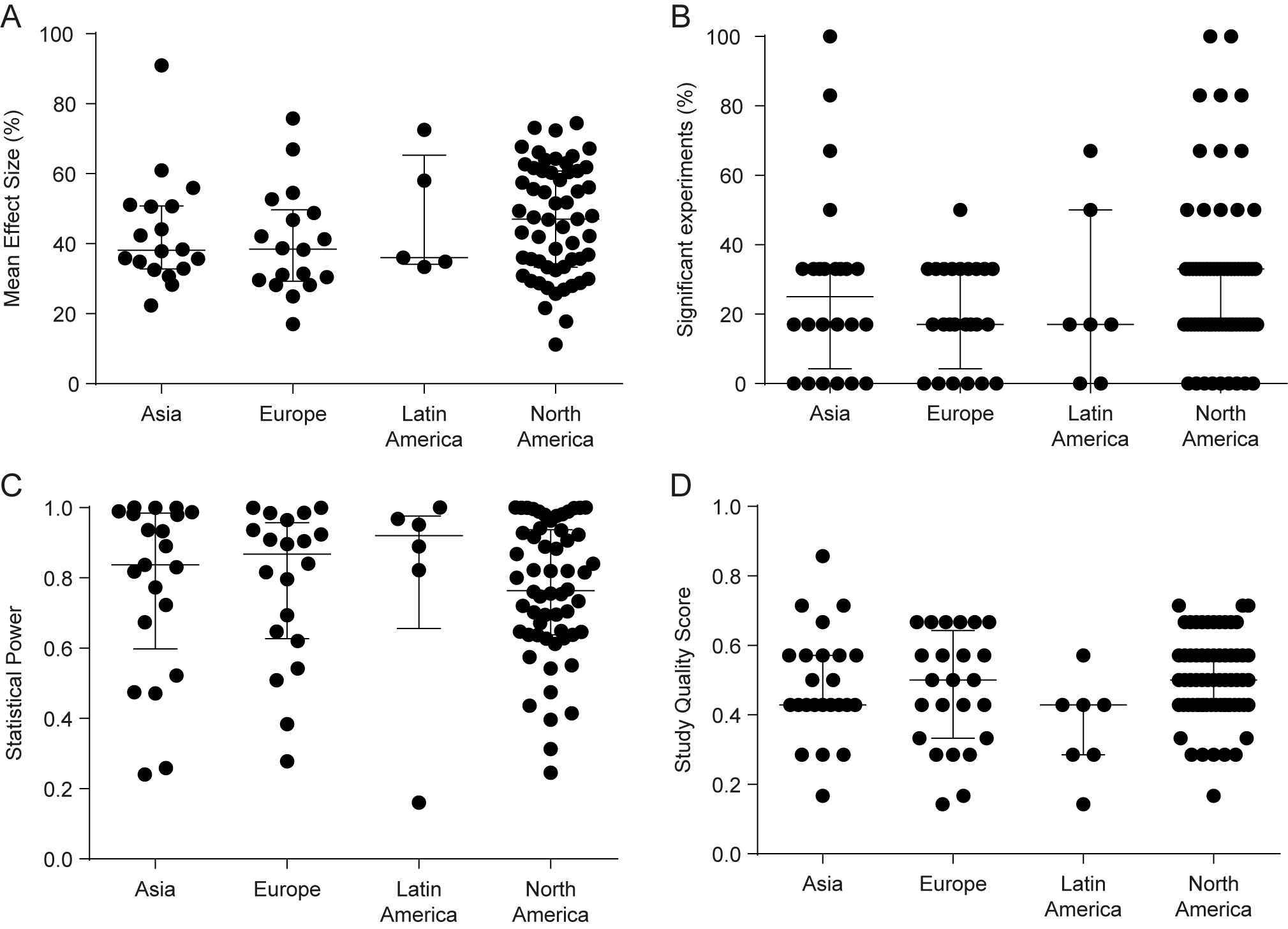

Supplement: S13 Fig — The region of origin of articles were defined according to the affiliation of the corresponding author (or authors) of each article. Line and whiskers express median and interquartile interval. (A) Distribution of mean effect size for significant results across regions of origin. One-way ANOVA p = 0.49. (B) Percentage of significant results per paper across regions of origin. One-way ANOVA p = 0.34 (C) Distribution of mean statistical power (upper-bound estimate) across regions of origin. One-way ANOVA p = 0.98. (D) Distribution of study quality scores across regions of origin. One-way ANOVA p = 0.11. According to Holm-Sidak correction for 17 article-level comparisons, none of the differences is significant. (TIF) [file pone.0196258.s014.tif]

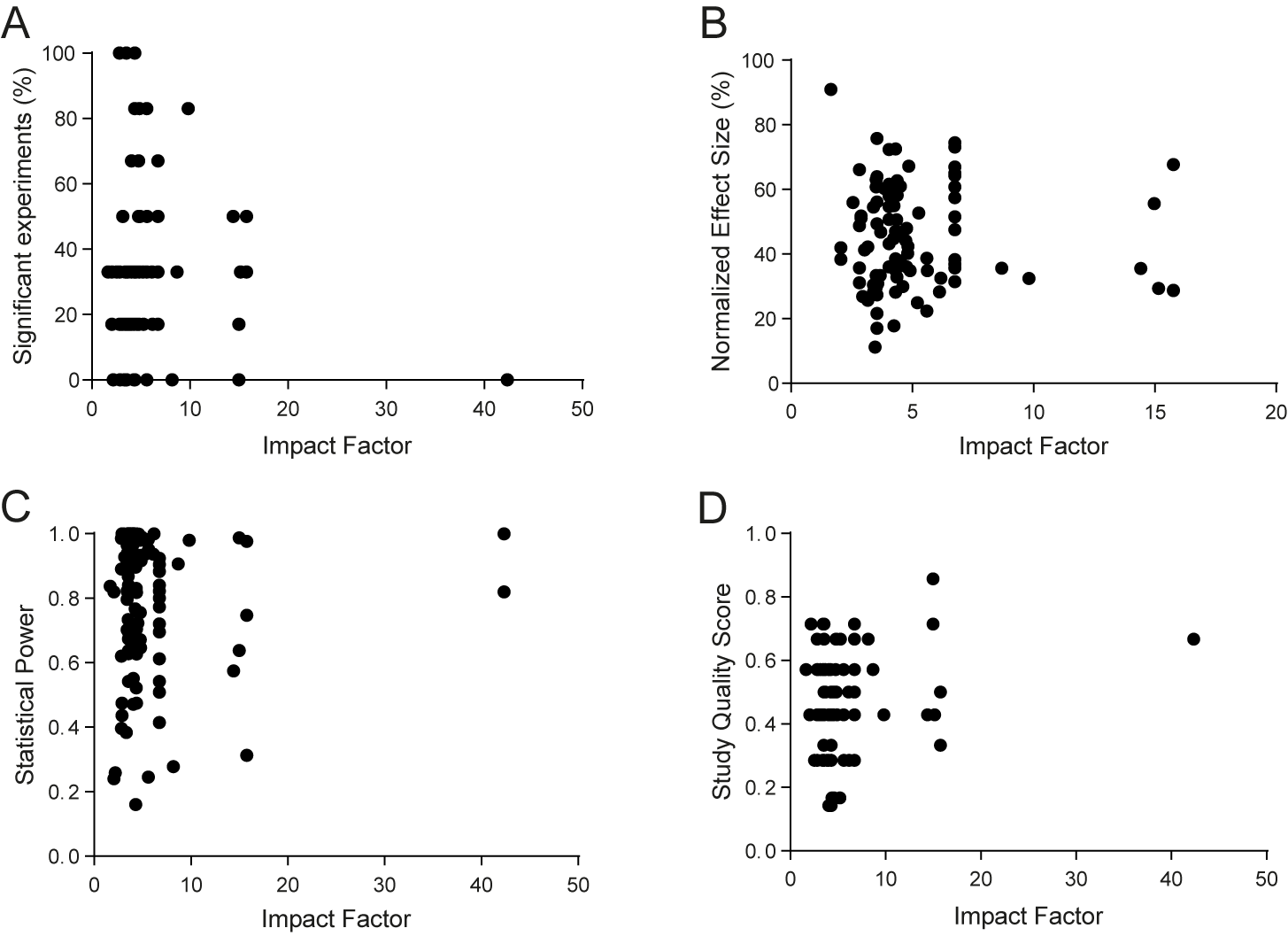

Supplement: S14 Fig — Impact factors were obtained from the 2013 Journal Citation Report. (A) Correlation between mean normalized effect size and impact factor. r = -0.05, p = 0.63 (n = 98). (B) Correlation between % of significant results per article and impact factor. r = -0.08, p = 0.37 (n = 121). (C) Correlation between mean statistical power (upper-bound estimate) and impact factor. r = 0.05, p = 0.62 (n = 104). (D) Correlation between study quality score and impact factor. r = 0.22, p = 0.01 (n = 121). According to Holm-Sidak correction for 8 article-level correlations, none of them are significant. (TIF) [file pone.0196258.s015.tif]
