# Supplementary material for: Effect size and statistical power in the rodent fear conditioning literature – A systematic review
Source: PLoS One. 2018 Apr 26;13(4):e0196258. doi: 10.1371/journal.pone.0196258 (PMC5919667; doi:10.1371/journal.pone.0196258)
Supplement: S1 Table — Volunteers were asked to judge each term on the left column as representing a strong, neutral or weak effect. Each term was given a score (strong, 2; neutral, 1; weak, 0) by each respondent and the mean score for each term (right column) was calculated based on an average of all 14 researchers. Single-measures intraclass correlation coefficient (reflecting agreement among researchers) was .234, while average-measures intraclass correlation coefficient (reflecting the aggregated reliability of the obtained means) was .839. Terms are ordered by score from weakest to strongest. (DOCX) [file pone.0196258.s016.docx]

| **Description Term** | **Strong (2)** | **Neutral (1)** | **Weak (0)** | **Score** |
| --- | --- | --- | --- | --- |
| **Less freezing** | 0 | 6 | 8 | 0.43 |
| **Lower freezing** | 0 | 7 | 7 | 0.50 |
| **Decrease** | 0 | 8 | 6 | 0.57 |
| **Reduction** | 0 | 8 | 6 | 0.57 |
| **More freezing** | 1 | 6 | 6 | 0.61 |
| **Deficit** | 2 | 6 | 6 | 0.71 |
| **Increase** | 1 | 8 | 5 | 0.71 |
| **Significantly more** | 0 | 10 | 4 | 0.71 |
| **Significantly shorter** | 0 | 10 | 4 | 0.71 |
| **Significantly smaller** | 0 | 10 | 4 | 0.71 |
| **Higher** | 2 | 7 | 5 | 0.79 |
| **Improved** | 2 | 7 | 5 | 0.79 |
| **Significantly effaced** | 1 | 9 | 4 | 0.79 |
| **Significantly higher** | 1 | 9 | 4 | 0.79 |
| **Significantly less** | 1 | 9 | 4 | 0.79 |
| **Significantly lower** | 1 | 9 | 4 | 0.79 |
| **Significant difference** | 1 | 10 | 3 | 0.86 |
| **Impairment** | 3 | 7 | 4 | 0.93 |
| **Significant effect** | 3 | 8 | 3 | 1.00 |
| **Significant impairment** | 4 | 7 | 3 | 1.07 |
| **Significant increase** | 4 | 7 | 3 | 1.07 |
| **Significantly enhanced** | 3 | 9 | 2 | 1.07 |
| **Significantly reduced** | 3 | 9 | 2 | 1.07 |
| **Enhanced** | 6 | 4 | 4 | 1.14 |
| **Significant decrease** | 4 | 8 | 2 | 1.14 |
| **Significant deficit** | 4 | 8 | 2 | 1.14 |
| **Significantly greater** | 4 | 8 | 2 | 1.14 |
| **Significant enhancement** | 5 | 7 | 2 | 1.21 |
| **Significant reduction** | 5 | 7 | 2 | 1.21 |
| **Disrupted** | 9 | 3 | 2 | 1.50 |
| **Clear decrease** | 12 | 1 | 1 | 1.79 |
| **Clear deficit** | 12 | 1 | 1 | 1.79 |
| **Marked decrease** | 12 | 1 | 1 | 1.79 |
