# Supplementary material for: Effect size and statistical power in the rodent fear conditioning literature – A systematic review
Source: PLoS One. 2018 Apr 26;13(4):e0196258. doi: 10.1371/journal.pone.0196258 (PMC5919667; doi:10.1371/journal.pone.0196258)
Supplement: S2 Table — Volunteers were asked to judge each term on the left column as representing similar means between both groups, a trend of difference or no information on the presence of a trend (neutral). Each term was given a score (trend, 2; neutral, 1; similar, 0) by each respondent and the mean score for each term (right column) was calculated based on an average of all 14 researchers. Single-measures intraclass correlation coefficient (reflecting agreement among researchers) was .597, while average-measures intraclass correlation coefficient (reflecting the aggregated reliability of the obtained means) was .962. Terms are ordered by score from most similar to most associated with a trend. (DOCX) [file pone.0196258.s017.docx]

| **Description Term** | **Trend (2)** | **Neutral (1)** | **Similar (0)** | **Score** |
| --- | --- | --- | --- | --- |
| **Comparable** | 0 | 0 | 13 | 0 |
| **Equal freezing** | 0 | 0 | 14 | 0 |
| **Equivalent** | 0 | 0 | 14 | 0 |
| **Same freezing** | 0 | 0 | 14 | 0 |
| **Similar** | 0 | 0 | 13 | 0 |
| **Did not differ** | 0 | 2 | 12 | 0.14 |
| **No change** | 0 | 2 | 12 | 0.14 |
| **No differences** | 0 | 2 | 12 | 0.14 |
| **Normal behavior** | 0 | 2 | 12 | 0.14 |
| **Undistinguishable** | 0 | 2 | 12 | 0.14 |
| **Did not affect** | 0 | 3 | 11 | 0.21 |
| **Did not interfere** | 0 | 3 | 11 | 0.21 |
| **No deficits** | 0 | 3 | 11 | 0.21 |
| **No effect** | 0 | 3 | 11 | 0.21 |
| **No variation** | 0 | 3 | 11 | 0.21 |
| **Not impaired** | 0 | 4 | 10 | 0.29 |
| **Not reduced** | 0 | 4 | 10 | 0.29 |
| **Failed to find differences** | 0 | 5 | 9 | 0.36 |
| **Did not find a significant effect** | 0 | 8 | 6 | 0.57 |
| **No significant alteration** | 3 | 3 | 8 | 0.64 |
| **No significant difference** | 3 | 3 | 8 | 0.64 |
| **Not significant** | 2 | 6 | 6 | 0.71 |
| **Not statistically significant** | 2 | 6 | 6 | 0.71 |
| **No reliable differences** | 6 | 3 | 5 | 1.07 |
| **Did not induce dramatic changes** | 7 | 3 | 4 | 1.21 |
| **Non-significant increase** | 9 | 3 | 2 | 1.50 |
| **Less freezing** | 11 | 3 | 0 | 1.79 |
| **Enhancement** | 12 | 2 | 0 | 1.86 |
| **Trended** | 13 | 0 | 1 | 1.86 |
